# Supplementary material for: Both age and social environment shape the phenotype of ant workers
Source: Sci Rep. 2023 Jan 5;13:186. doi: 10.1038/s41598-022-26515-1 (PMC9814961; doi:10.1038/s41598-022-26515-1)
Supplement: Supplementary file 1 — Supplementary Information. [file 41598_2022_26515_MOESM1_ESM.docx]

Electronic supplementary Material

Both age and social environment shape the phenotype of ant workers.

Martin Quque*^1^, Charlotte Brun^1,2^, Claire Villette^3^, François Criscuolo^1^, Cédric Sueur^1,4^, Dimitri Heintz^3,ǂ^, Fabrice Bertile^1,2, ǂ^

^1^ Université de Strasbourg, CNRS, IPHC UMR 7178 – 23 rue du Loess, 67037 Strasbourg Cedex 2, France

^2^ Infrastructure Nationale de Protéomique ProFI – 25 rue Becquerel, 67037 Strasbourg Cedex 2, France

^3^ Plant Imaging & Mass Spectrometry (PIMS), Institut de biologie moléculaire des plantes, CNRS, Université de Strasbourg, 12 rue du Général Zimmer, 67084 Strasbourg, France.

^4^ Institut Universitaire de France, 1 rue Descartes, 75231 Paris Cedex, France

ǂ Share senior authorship of the paper

***** Correspondence: [martin.quque@iphc.cnrs.fr](mailto:martin.quque@iphc.cnrs.fr) - +33 388 106 951

**ESM1 – Workflow of the joint proteomics-metabolomics analysis**

**[below] Figure S1. Workflow from sample preparation to data analysis.** **A)** After collecting freshly mated queens of black garden ants (*Lasius niger*) in the field, we let new colonies to settles down for one month, and then we collected young foragers (*Y.F*) and young nest workers (*Y.NW*). From this date, larvae were removed and 11 months later, we collected workers again: old foragers (*O.F*) and old nest workers (*O.NW*). Samples were stored intact at -80°C and processed at the same time in the mass spectrometry analyses. **B)** The proteomics (at left in green) and metabolomics (at right in red) protocols were run in parallel as shown in this figure and detailed in the “Material and Methods” section. **C)** We detected 1719 proteins and 712 metabolites among the four experimental groups. An analyte (metabolite or protein) was considered as ‘*present in all castes*’ when found in at least 3 samples per caste and as ‘*absent in at least one caste*’ when not found in any sample of one or more caste. All other analytes were *not selected* for further analysis. An *annotated* analyte means that a name was automatically attributed by querying spectral databases. Further details in the main text.


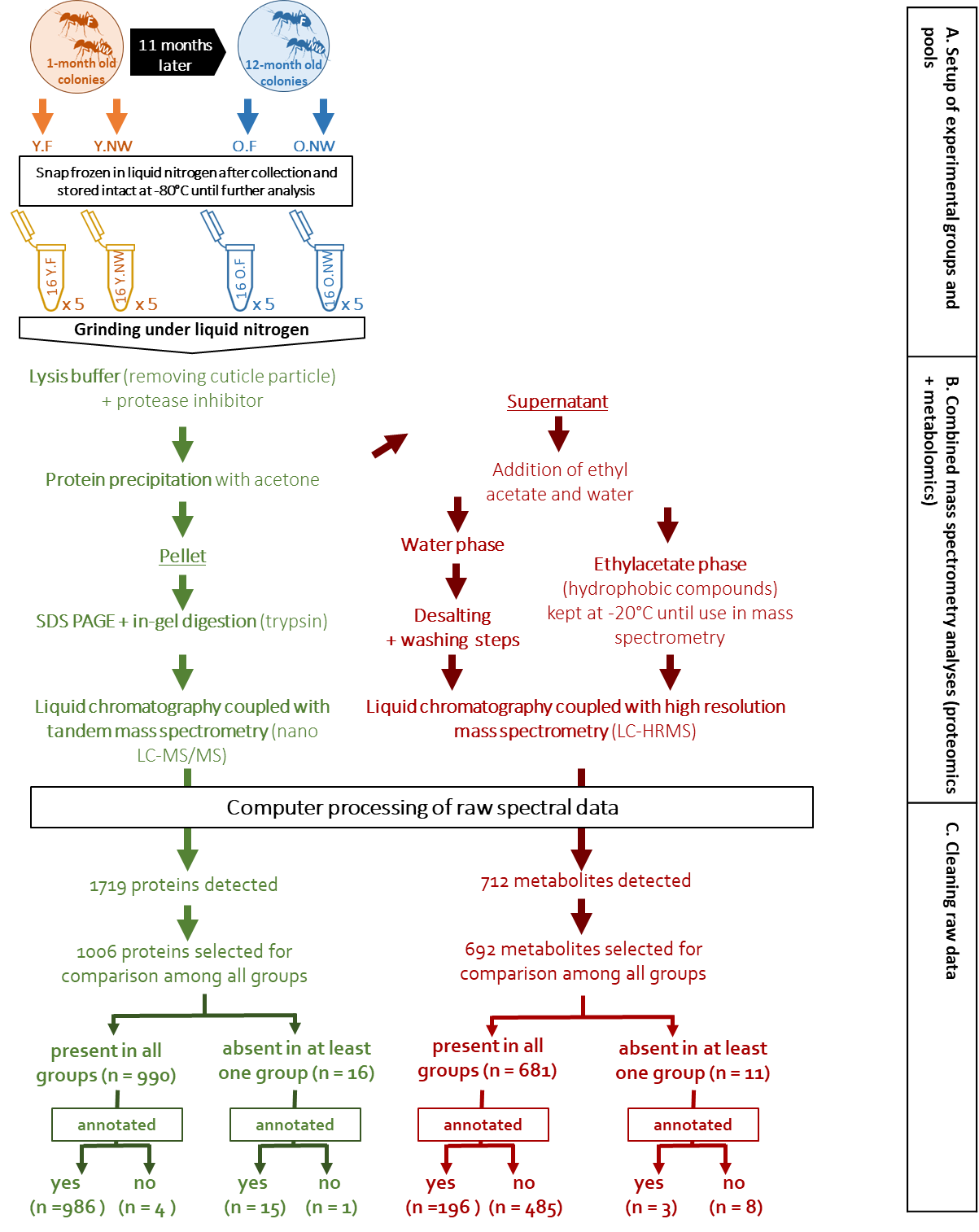


# ESM2 – Functional enrichment analysis


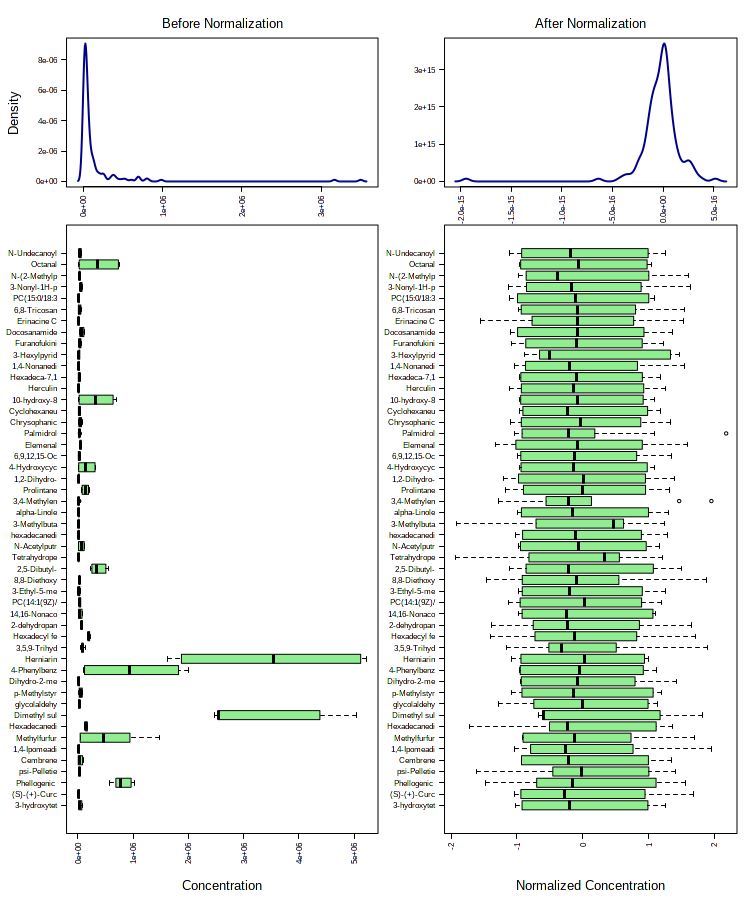
To understand the biological meaning of proteomics and metabolomics profiles, we ran a functional enrichment analysis. This method compares the relative expression of metabolic pathways, according to the amount of molecules involved in them, among experimental groups. Thus, we aimed to retrieve a functional profile of our experimental groups.

## Figure S2 Normalization of our metabolomics dataset. Figures provided by MetaboAnalyst after normalization through the ‘auto-scaling’ option (mean-centred and divided by the standard deviation of each variable).


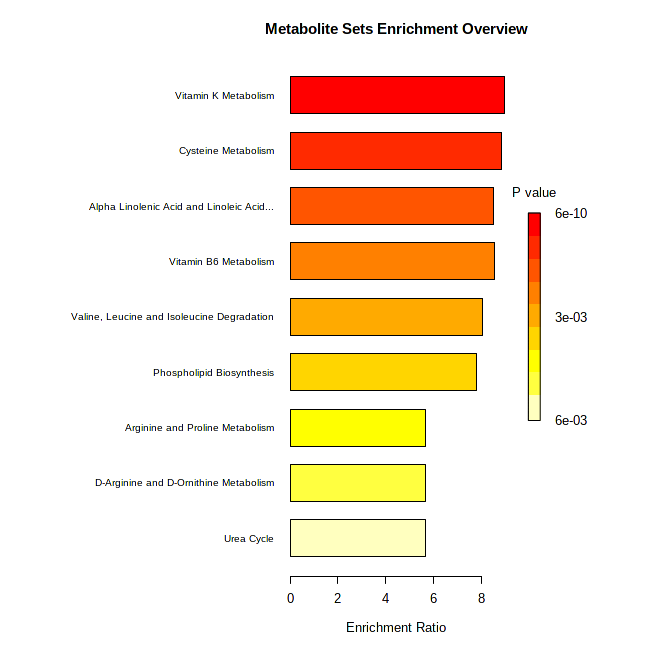
We used the online platform MetaboAnalyst (v. 5.0, www.metaboanalyst.ca; Pang et al. 2021) and ran an automated metabolite set enrichment analysis (aka. MSEA). Metabolites are usually assessed individually for their significance under the study conditions. On the contrary, MSEA directly evaluates the significance of a set of functionally related metabolites. During the quantitative enrichment analysis, the data were normalized with the ‘auto-scaling’ option (see **Figure S2** above). We queried both the Small Molecule Pathway Database (SMPDB) and the Kyoto Encyclopedia of Genes and Genomes (KEGG) since they provide complementary pathways. We considered all molecules in these databases that had at least two entries. However, the databases used by MetaboAnalyst are mainly derived from human studies. This led to the fact that among 196 annotated metabolites kept for the characterization of the four experimental worker groups, only 53 (27.04%) were recognized by the platform. The databases used by the platform do not sufficiently cover our dataset to fully depict its diversity. The outcomes of the analysis can be found below.

**Figure S3 Metabolite Set Enrichment using SMPDB**


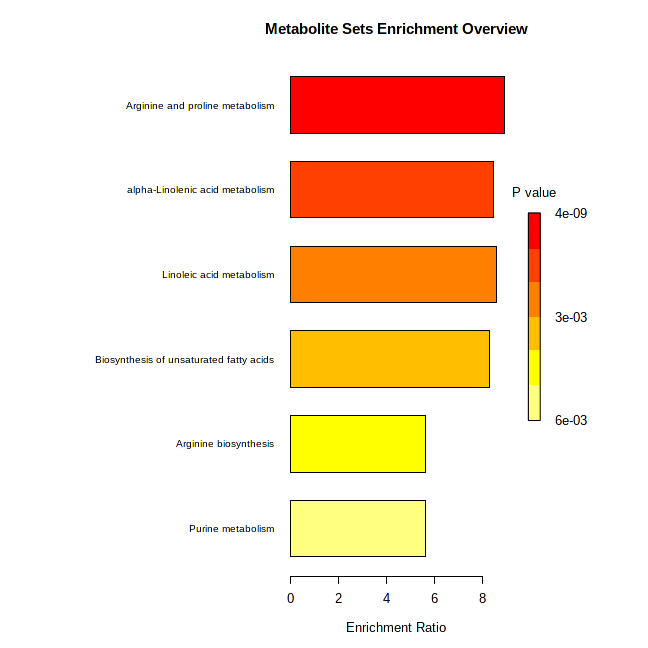


**Figure S4 Metabolite Set Enrichment using KEGG**

We can notice overlaps between both databases. The quantitative enrichment analyses mostly highlighted pathways linked to amino acids (arginine, valine, leucine), linoleic acids, and vitamins. As we can notice from the comparison with the methodology used in the main text, using this approach would have led to missing many biological functions (e.g., oxidative status, digestive function, cancer-related molecules). As discussed above, this is probably linked to the fact that those databases are intended for studies on humans.
